# Sustained Improvement in Clinical Preventive Service Delivery Among Independent Primary Care Practices After Implementing Electronic Health Record Systems

**DOI:** 10.5888/pcd10.120341

**Published:** 2013-08-01

**Authors:** Jason J. Wang, Kimberly M. Sebek, Colleen M. McCullough, Sam J. Amirfar, Amanda S. Parsons, Jesse Singer, Sarah C. Shih

**Affiliations:** Author Affiliations: Kimberly M. Sebek, Colleen M. McCullough, Sam C. Amirfar, Amanda S. Parsons, Jesse Singer, Sarah C. Shih, New York City Department of Health and Mental Hygiene, Primary Care Information Project, Queens, New York.

## Abstract

**Introduction:**

Studies showing sustained improvements in the delivery of clinical preventive services are limited. Fewer studies demonstrate sustained improvements among independent practices that are not affiliated with hospitals or integrated health systems. This study examines the continued improvement in clinical quality measures for a group of independent primary care practices using electronic health records (EHRs) and receiving technical support from a local public health agency.

**Methods:**

We analyzed clinical quality measure performance data from a cohort of primary care practices that implemented an EHR at least 3 months before October 2009, the study baseline. We assessed trends for 4 key quality measures: antithrombotic therapy, blood pressure control, smoking cessation intervention, and hemoglobin A1c (HbA1c) testing based on monthly summary data transmitted by the practices.

**Results:**

Of the 151 practices, 140 were small practices and 11 were community health centers; average time using an EHR was 13.7 months at baseline. From October 2009 through October 2011, average rates increased for antithrombotic therapy (from 58.4% to 74.8%), blood pressure control (from 55.3% to 64.1%), HbA1c testing (from 46.4% to 57.7%), and smoking cessation intervention (from 29.3% to 46.2%). All improvements were significant.

**Conclusion:**

During 2 years, practices showed significant improvement in the delivery of several key clinical preventive services after implementing EHRs and receiving support services from a public health agency.

## Introduction

Since the publication of a report by McGlynn et al (1), delivery rates of clinical preventive services are increasing, but slowly. The 2012 State of Health Care Quality report from the National Committee for Quality Assurance (NCQA) shows small improvements during the past 5 years in recommended preventive services for related cardiovascular measures, which have increased from 1 to 2 percentage points per year (2). Efforts to drive improvements include the use of health information technology (health IT), transformation of primary care practices into patient-centered medical homes, and payment reform (3–9).

Although several of these initiatives have yielded financial and clinical benefits, most have been demonstrated in closed, integrated delivery systems (10,11). Demonstrations in smaller independent settings have yet to produce consistent evidence that improving the quality of care is possible, which is disconcerting given that most ambulatory office visits in the United States still occur in practices operating with fewer than 10 physicians (12). Concerns continue that small independent practices are less likely to have programs to leverage advanced information systems for improving care coordination and disease management, potentially reducing hospitalizations or emergency department visits (12–15). Furthermore, small practices are unable to pool resources as larger integrated systems do to support their delivery infrastructure and dedicate nonphysician staff to help act on missed preventive services, because these require significant financial investments (16). Small practices also lag behind large practices in the adoption of health IT systems, and this gap continues to widen (17).

In expanding the public health role with primary care, the New York City Department of Health and Mental Hygiene (NYC DOHMH) created a new bureau, the Primary Care Information Project (PCIP), whose mission is to improve the delivery of clinical preventive services through the use of data-driven approaches for improving population health (18). Supported through a mix of federal, state, city, and private funding, PCIP has assisted 3,000 primary care providers at more than 500 independent practices to adopt electronic health record (EHR) systems and guided practices in transformation activities and participation in incentive programs (18). Collectively, these providers represent approximately 25% of New York City’s primary care providers and serve more than 2.6 million patients. Practices participating with PCIP are given tools to track and trend their panel of patients’ health across the city’s health agenda, Take Care New York (TCNY) (19), which includes 10 areas of actionable recommendations for patients, health care providers, and community partners. This study describes 2-year trends in performance of 4 key clinical quality measures, a subset of the 10 areas of health recommended by TCNY in which improvement in these measures has the highest potential for reducing preventable death in New York City and in the United States (20).

## Methods

Practices were included if they adopted the PCIP subsidized eClinicalWorks EHR system at least 3 months before the baseline data analysis period (October 2009). Practices were excluded if data were not transmitted at any of the 3 data analysis periods: October 2009 (T1), October 2010 (T2), and October 2011 (T3). Community health centers (CHCs) were defined as any PCIP practice that was either a federally qualified health center, a New York State-defined diagnosis and treatment center, or had unusual special needs such as serving a foster care, mentally disabled, or other special population. All other practices were classified as small practices (SPs).

Data shown in the analysis were derived from practice EHRs that automatically transmit quality measurement data monthly to PCIP. From these transmissions, averages of practice performance data were assembled from October 2009 through October 2011. Four key quality measures of relevance to the TCNY agenda were included in the analytical data set: antithrombotic therapy among patients aged 18 years or older with ischemic vascular disease or aged 40 years or older with diabetes; blood pressure (BP) control among patients aged 18 to 75 years with essential hypertension and no diagnosis of ischemic vascular disease or diabetes; hemoglobin A1c (HbA1c) testing in the past 6 months among patients aged 18 to 75 years with diabetes; and smoking cessation intervention in the past 12 months among patients aged 18 years or older who were “current smokers” (Table 1). These measures are similar to National Quality Forum endorsed measures (no. 0631 [antithrombotic therapy], no. 0018 [BP control], no. 0057 [HbA1c testing], and no. 0028 [smoking cessation intervention]) (21), with the exception of age ranges and the fact that patients must have had at least 1 office visit with the primary care practice to be eligible. No other rules were applied for patient inclusion to the denominator. Additional practice and provider variables included number of encounters and number of unique patients seen per month, practice location, number of providers, number of practice sites, and practice-reported percentage of patients on Medicaid or self-insured. Practice date of EHR implementation was obtained from a customer relationship management database maintained by PCIP staff.

**Table 1:** Description of Quality Measures, New York City Primary Care Practices (N = 151)

Measure	Eligible Patient (Denominator)	Patient Goal (Numerator)
**HbA1c testing**	Patients aged 18 to 75 years with diabetes	HbA1c test recorded in the past 6 months
**Antithrombotic therapy**	Patients aged 18 years or older with ischemic vascular disease or aged 40 years or older with diabetes	Taking antithrombotic/other antithrombotic therapy
**Blood pressure control**	Patients aged 18 to 75 years with hypertension and no diagnosis of ischemic vascular disease or diabetes	Systolic blood pressure <140 mm Hg and diastolic blood pressure <90 mm Hg
**Smoking cessation intervention**	Patients aged 18 years or older with a “current smoker”^a^ smoking status	Smoking cessation intervention (prescription or counseling) received in the past 12 months

Abbreviation: HbA1c, hemoglobin A1c.

a A “current smoker” was identified through the most recent documented status located in a structured form in the electronic health record. Providers selected either “current smoker,” “former smoker,” or “never smoker.”

All statistical analyses were conducted using SAS version 9.2 (22). Comparisons between groups of practices, time points, or trends were considered significant if the observed *P* value for a statistical test was less than .05. Practice characteristics were measured at the baseline period (October 2009). Chi-square tests, *t* tests and nonparametric tests were used to compare characteristics between SPs and CHCs.

Trend graphs were generated for each quality measure over time and show average monthly practice rates. Separately, a comparison of practice level performance rates on each of the 4 quality measures was conducted for 3 points: October 2009 (T1), October 2010 (T2), and October 2011 (T3); and by practice characteristics. Generalized estimating equation models with logistic link function were used to analyze the association between time and practice characteristics and performance on the selected quality measures.

## Results

Of the 550 independent primary care practices assisted by PCIP with EHR implementation, 151 (140 SPs and 11 CHCs) met all of the inclusion criteria for this analysis; 309 practices were excluded because they had not been using their systems for 3 months by October 2009, and 90 additional practices were excluded because they did not transmit data for all 3 data analysis points. A comparison of practice characteristics between those included in this analysis (n = 151) and those not included (n = 399) did not show any differences, with the exception of time using EHR.

Practices included in the trend analysis had an average of 925 encounters or 742 unique patients per month (Table 2). Practice characteristics, including patient distribution by borough, number of practice sites, and the length of time using an EHR system, were similar between SP and CHC groups. SPs differed from CHCs by number of providers the practice employed (2.6 vs 29.3, *P* = .03) and practice-reported percentage (20% or higher) of Medicaid or self-insured patients seen by the practice (29.3% vs 81.8%, *P* < .001).

**Table 2:** Practice Characteristics of Small Practices (SPs) and Community Health Centers (CHCs) at Baseline, New York City, October 2009

Characteristic	Mean Practice Value		
	All Practices (n = 151)	SPs (n = 140)	CHCs (n = 11)
No. of sites	1.4	1.2	3.1
No. of providers	4.5	2.6	29.3^a^
No. of encounters per month	925	749	3,169
No. of unique patients per month	742	612	2,392
% of Medicaid/self-insured ≥20 (practice-reported)	33.1	29.3	81.8^b^
Months using EHR up to October 2009	13.7	13.8	13.0
Bronx	12.6	12.9	9.1
Brooklyn	33.1	34.3	18.2
Manhattan	31.1	29.3	54.6
Queens	18.5	18.6	18.2
Staten Island	4.6	5	0

Abbreviation: EHR, electronic health record.

a *P* = .03

b *P* < .001.

During the 2-year period, practice performance on all 4 quality measures exhibited an upward trend (Figure). Rates increased for antithrombotic therapy (58.4% to 74.8%, *P* < .001, standard deviation [SD] =19.7%), BP control (55.3% to 64.1%, *P* = .001, SD = 16.9%), HbA1c testing (46.4% to 57.7%, *P* = .007, SD = 29.6%), and smoking cessation intervention (29.3% to 46.2%, *P* < .001, SD = 27.1%). All improvements between baseline and T3 were significant. Improvements were higher in the second time interval (T3 vs T2) than in the first (T2 vs T1) for blood pressure control, HbA1c testing, and smoking cessation intervention.

**Figure:**
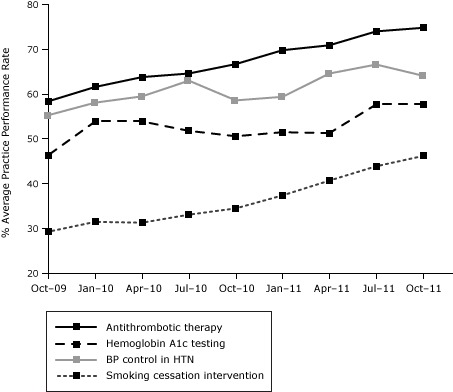
Trend of individual quality measures over 3 periods: October 2009 (T1), October 2010 (T2), and October 2011 (T3). **Table:** 

Period	% Average Practice Performance Rate			
	Antithrombotic Therapy	HbA1c Testing	Blood Pressure Control in Hypertension	Smoking Cessation Intervention
**October 2009**	58.4	46.4	55.3	29.3
**January 2010**	61.6	53.9	58.1	31.5
**April 2010**	63.8	53.9	59.5	31.3
**July 2010**	64.6	51.8	63.0	33.1
**October 2010**	66.7	50.6	58.6	34.5
**January 2011**	69.8	51.5	59.4	37.4
**April 2011**	70.9	51.3	64.6	40.7
**July 2011**	74.0	57.7	66.6	43.9
**October 2011**	74.8	57.7	64.1	46.2

Improvement was consistently observed on all measures across practices over time. Practice characteristics, such as organization type and number of providers, were generally not associated with performance at each time point across the 4 measures. Time using the EHR (measured as months since EHR implementation) was the only factor consistently associated with improved performance, a finding that was also confirmed by a multivariate model adjusted for various practice characteristics: antithrombotic therapy (odds ratio [OR] = 1.03; 95% confidence interval [CI], 1.02–1.04); BP control (OR = 1.006; 95% CI, 1.00–1.01); HbA1c testing (OR = 1.03; 95% CI, 1.01–1.04); and smoking cessation intervention (OR = 1.04; 95% CI, 1.02–1.05) (Table 3). Practices with higher rates of self-reported Medicaid/uninsured populations achieved more significant improvement for smoking cessation intervention (OR = 1.78; 95% CI, 1.13–2.82)] than practices serving smaller Medicaid populations.

**Table 3:** Association Between EHR Use and Practice Characteristics and Performance, by Quality Measure, New York City, 2009–2011

Practice Characteristics	Quality Measure			
	Antithrombotic Therapy	BP Control	HbA1c Testing	Smoking Cessation Intervention

	Odds Ratio (95% Confidence Interval)			
Months since implementing EHR	1.03 (1.02–1.04)	1.006 (1.00–1.01)	1.03 (1.01–1.04)	1.04 (1.02–1.05)
CHC	0.84 (0.43–1.63)	0.56 (0.38–0.81)	1.13 (0.32–3.98)	0.58 (0.28–1.20)
Months since EHR*CHC^a^	1.003 (0.98–1.02)	1.01 (1.002–1.02)	1.02 (0.99–1.06)	1.006 (0.96–1.05)
Medicaid/self-pay ≥20%	1.21 (0.89–1.66)	1.08 (0.89–1.31)	1.21 (0.83–1.78)	1.78 (1.13–2.82)
Practice has >1 provider	1.24 (0.83–1.85)	0.93 (0.76–1.13)	1.12 (0.74–1.70)	0.70 (0.45–1.08)

Abbreviations: EHR, electronic health record; BP, blood pressure; HbA1c, hemoglobin A1c; EHR, electronic health record; CHC, community health center.

^a^ Interaction between months since implementing an EHR and CHC status.

## Discussion

Two-year trends of 151 independent practices showed significant gains on 4 key quality measures: antithrombotic therapy, BP control, HbA1c testing, and smoking cessation intervention. Our findings suggest that independent SPs and CHCs, with assistance from a community EHR extension program such as PCIP, can achieve clinical quality gains similar to those observed in larger, well-resourced integrated delivery systems (2). Our findings are relevant to independent practices serving resource-challenged urban areas.

Several reports in the literature indicated that practice size is associated with higher quality of care (23,24), so we also conducted analyses to ascertain whether the level of improvement differed across practice characteristics, such as whether CHCs experienced greater improvements than SPs. Of the practice characteristics we analyzed, none accounted for consistent differences in the increases observed with the exception of duration using an EHR.

Previous reports from this population suggest that short-term improvements are possible (25,26); in this study, we observed increases of several percentage points per year, suggesting that long-term improvement can also occur. This continued progress supports the idea that urban independent practices can drive long-term improvements in population health, a finding that is promising for inner-city independent practices like those served by PCIP, because they see an above-average number of patients who are uninsured and who have more severe health issues (17). At the same time, policy makers or stakeholders looking for instant quality gains should be cautious to expect early returns post-EHR implementation, because the larger increases in improvement were seen in the latter time period — between October 2010 and 2011 — when practices had been using the EHR for 25 months or more.

Our study has limitations. It was limited to the 151 practices where we received consistent quality measurement data over the 2-year period and therefore did not include all practices that joined PCIP and received similar support. Practices included in the study were generally earlier adopters of EHR, and we could not infer whether the performance trend would be similar or dissimilar to that of practices who were later adopters. We did not include specialties or other facilities that may interact with patients.

Providers working with PCIP represent a group of EHR users who have received varied assistance from PCIP staff, including training and guidance on quality improvement strategies, technical support on EHR software (upgrades, patches, and configuration), and connection for health information exchange. This type of support has been shown to positively affect the transition to an EHR system (27,28). A separate study using claims-based data suggests that improvement on quality measures can be achieved for practices that have used technical assistance (29). By the end of the analysis period in 2011, practices had been exposed to several environmental changes that were not measured or tested in this study. These include the introduction of the Centers for Medicaid and Medicare Services Meaningful Use incentives, New York State Medicaid incentives for practices achieving recognition for patient-centered medical homes, and monthly dashboards from PCIP for trending performance on selected EHR use and quality measures. Whether the introduction of these programs had an affect on the observed quality trends is unknown.

Improvement resulting from better documentation alone in the EHR was not tested in this study. However, we are aware that documentation and work-flow variability can result in underreporting of practice performance for some measures and not others (30). For example, HbA1c testing in patients with diabetes requires laboratory values to be returned through an electronic interface or manually entered into the patient’s record by the provider or practice staff. Practice rates on laboratory tests in which an electronic laboratory interface was not available and the practice does not routinely enter results into the patient’s record will be underreported, because EHR quality measurement programming will not detect information in scanned documents or faxes. Smoking cessation intervention is subject to appropriate documentation of counseling or prescription of smoking cessation aids; providers may not have instituted appropriate workflows to capture counseling conversations in the appropriate section of the EHR, thus underreporting the delivery of cessation intervention. In the example of antithrombotic therapy, gains in performance may be explained by improved documentation of over-the-counter medications (eg, aspirin) while continued trends represent better attention to this preventive service.

Broad adoption of EHR systems, along with technological advances and more experience with measurement using data from electronic records, may further drive improvements in primary care. However, it is promising to note that in our study, performance among independent practices was similar to or in some cases, exceeded, that of larger integrated systems. For instance, in 2011, PCIP averaged 64% on the BP control measure. For that measure in the same year, NCQA reported that nationally, commercial health maintenance organizations were averaging 65% and commercial and Medicare PPOs and Medicaid HMOs were averaging 58% to 60% (2). Both providers and policy makers should be encouraged by this indicator that independent primary care physicians can keep pace with their peers who work in integrated systems and can continue to improve, post-EHR adoption.

In our experience, the amount of support providers need to realize quality gains varies widely. Practices may require anywhere from 2 to 10 onsite visits in a year from a midlevel clinical quality specialist (29). These resources could be further sustained through local programs or through stakeholder support, such as payers or employer-based initiatives. Continued federal incentives and new payment models from Centers for Medicare and Medicaid Services may help stimulate independent primary care practices to get the most from health IT as an investment to improve health care and focus on patient-centered, outcomes-driven care and coordination.
